# Enhanced Replication of Hepatitis E Virus Strain 47832c in an A549-Derived Subclonal Cell Line

**DOI:** 10.3390/v8100267

**Published:** 2016-09-29

**Authors:** Mathias Schemmerer, Silke Apelt, Eva Trojnar, Rainer G. Ulrich, Jürgen J. Wenzel, Reimar Johne

**Affiliations:** 1 Institute of Clinical Microbiology and Hygiene, 93053 Regensburg, Germany; Mathias.Schemmerer@klinik.uni-regensburg.de (M.S.); Juergen.Wenzel@klinik.uni-regensburg.de (J.J.W.); 2 German Federal Institute for Risk Assessment, Department of Biological Safety, 12277 Berlin, Germany; Silke.Apelt@bfr.bund.de (S.A.); Eva.Trojnar@bfr.bund.de (E.T.); 3 Friedrich-Loeffler-Institut, Institute for Novel and Emerging Infectious Diseases, 17493 Greifswald-Insel Riems, Germany; Rainer.Ulrich@fli.bund.de

**Keywords:** hepatitis E virus, cell culture, A549, CEACAM, syndecan

## Abstract

Hepatitis E virus (HEV) is a human pathogen with increasing importance. The lack of efficient cell culture systems hampers systematic studies on its replication cycle, virus neutralization and inactivation. Here, several cell lines were inoculated with the HEV genotype 3c strain 47832c, previously isolated from a chronically infected transplant patient. At 14 days after inoculation the highest HEV genome copy numbers were found in A549 cells, followed by PLC/PRF/5 cells, whereas HepG2/C3A, Huh-7 Lunet BLR and MRC-5 cells only weakly supported virus replication. Inoculation of A549-derived subclone cell lines resulted in most cases in reduced HEV replication. However, the subclone A549/D3 was susceptible to lower virus concentrations and resulted in higher virus yields as compared to parental A549 cells. Transcriptome analysis indicated a downregulation of genes for *carcinoembryonic antigen-related cell adhesion molecules* (*CEACAM*) *5* and *6*, and an upregulation of the *syndecan 2* (*SDC2*) gene in A549/D3 cells compared to A549 cells. However, treatment of A549/D3 cells or A549 cells with CEACAM- or syndecan 2-specific antisera did not influence HEV replication. The results show that cells supporting more efficient HEV replication can be selected from the A549 cell line. The specific mechanisms responsible for the enhanced replication remain unknown.

## 1. Introduction

Hepatitis E virus (HEV) is the causative agent of acute hepatitis in humans. It causes worldwide an estimated 20 million cases and 56,000 deaths every year [1]. Most of the cases occur in developing countries where the virus is mainly transmitted by fecally contaminated drinking water. However, hepatitis E cases are also increasingly recognized in industrial countries where HEV is mostly zoonotically transmitted from pigs and wild boars [2]. In addition, chronic HEV infections of immunosuppressed transplant patients, which may cause liver cirrhosis, have been increasingly reported [3].

HEV particles are icosahedral and non-enveloped, although recent analyses of cell culture-derived HEV suggest the presence of an additional enveloped particle fraction [4]. The HEV genome consists of a single-stranded RNA with positive polarity. It contains the open reading frame (ORF) 1 encoding a non-structural polyprotein, ORF2 encoding the capsid protein and ORF3 encoding a small phosphoprotein. Four main human-pathogenic genotypes of HEV have been defined. Genotypes 1 and 2 exclusively infect humans, whereas genotypes 3 and 4 are zoonotic with main reservoirs in domestic pigs and wild boars [2].

The knowledge on the HEV replication cycle is limited, mainly because of the inefficiency of current cell culture models for HEV propagation. In addition, systematic studies on HEV neutralization and inactivation are hampered by the lack of cell culture-based infectivity assays [5]. Although many publications report the successful isolation of HEV using the human liver cell lines HepG2/C3A and PLC/PRF/5 or the human lung carcinoma cell line A549, virus replication usually progresses very slowly and infection with low amounts of virus results in failure of HEV replication [4,5,6,7,8]. The use of novel cell lines has been recently described [9,10,11], but their broad applicability and robustness remains to be proven. In addition, more efficiently replicating virus strains have been described [8,12,13]. One of them, designated as strain 47832c, has been recently isolated from a chronically infected transplant patient in Germany [8].

The aim of this study was to improve the cell culture replication of a cell culture-adapted HEV strain by testing different established cell lines as well as newly generated subclonal cell lines. Cell lines that are more susceptible to HEV infection should be characterized in more detail in order to understand the underlying mechanisms. The study should help to provide efficient HEV cell culture systems, which are urgently needed to enable investigation of HEV replication, neutralization and inactivation.

## 2. Materials and Methods

### 2.1. Cells and Viruses

The human liver carcinoma cell lines PLC/PRF/5 (ATCC CRL-8024; LGC Standards GmbH, Wesel, Germany), HepG2/C3A (ATCC CRL-10741; LGC Standards GmbH), Huh-7 Lunet BLR (kindly provided by Prof. Dr. Ralf Bartenschlager, University of Heidelberg, Germany), the fetal lung cell line MRC-5 (ATCC CCL-171; LGC Standards GmbH) as well as the human lung carcinoma cell line A549 (ATCC CCL-185; LGC Standards GmbH) were used. The HEV genotype 3 strain 47832c was originally isolated from a chronically infected transplant patient by inoculation onto A549 cells [8]. This strain has been shown to carry an insertion within the hypervariable genome region of the ORF1, which is derived from the 3′-region of this ORF. An A549 cell line persistently infected with this HEV strain has been established, which continuously releases HEV into the cell culture medium [8].

### 2.2. Generation of Clonal Cell Lines from A549 Cell Culture

A549 cells grown in a T25 flask were detached from the surface using trypsin and subsequently diluted to a concentration of 10 cells per mL in minimum essential medium (MEM) Eagle supplemented with 1% non-essential amino acids, 1% glutamine, 0.5% gentamicin and 10% fetal calf serum (FCS; all cell culture media by PAN Biotech GmbH, Aidenbach, Germany). The cells were seeded into a 96-well plate (100 µL per well) and incubated at 37 °C and 5% CO_2_ in a humidified incubator. The wells were regularly checked by light microscopy and only wells showing the growth of a single cellular clone were chosen for subsequent experiments. The culture supernatant was replaced weekly by fresh medium as above. After generation of a complete monolayer, the cells were trypsinized and grown successively in 24-well plates, 6-well plates and T25 flasks (Orange Scientific, Braine-l’Alleud, Belgium).

### 2.3. Virus Stock Preparation

The A549 cell line persistently infected with the HEV strain 47832c was grown in T25 flasks in MEM Eagle medium complemented with 1% non-essential amino acids, 1% glutamine, 1% gentamicin and 2% FCS at 37 °C and 5% CO_2_ in a humidified incubator. After seven days of incubation, the supernatant was removed, centrifuged to remove cells and stored at −80 °C. The cells were split 1:2 and grown for another seven days until collection of supernatant as above. The stored supernatants were tested by HEV-specific quantitative reverse transcription polymerase chain reaction (RT-qPCR) as described below and those containing high amounts of HEV RNA were pooled.

### 2.4. HEV Inoculation of Cell Cultures

HEV inoculation trials have been performed in two different laboratories under slightly different conditions. The initial tests with several different established cell lines were performed in laboratory 1 (Institute of Clinical Microbiology and Hygiene, Regensburg, Germany) and cells were grown in T12.5 flasks in MEM Eagle medium complemented with 1% non-essential amino acids, 1% glutamine, 1% gentamicin, 10 mM MgCl_2_ and 10% FCS at 37 °C and 5% CO_2_ in a humidified incubator. The cultures were grown for two weeks (with complete medium replacement every three or four days) prior to infection. All inoculation experiments were performed in triplicates. Cells were inoculated with supernatant containing the HEV strain 47832c (8.0 × 10^6^ genome copies/mL; 250 µL/flask for infection) and incubated at room temperature for 1 h. The virus suspension was replaced by fresh medium as above and cells were incubated at 34.5 °C and 5% CO_2_. Medium was replaced on day 1, 4, 7, 11 and 14 post inoculation (p.i.) as described above and aliquots were stored for analysis at −80 °C.

All other tests were performed in laboratory 2 (Federal Institute for Risk Assessment, Berlin, Germany) under conditions as described below. For comparison of subclonal cell lines, 24-well plates (RT-qPCR analysis) or 96-well plates (immunofluorescence analysis) were used. In these cases, cells were grown for seven days in MEM Eagle medium complemented with 1% non-essential amino acids, 1% glutamine, 0.5% gentamicin and 10% FCS at 37 °C and 5% CO_2_ in a humidified incubator. Thereafter, the culture supernatant was exchanged with fresh medium and the cells were incubated for additional three days as above. The confluent cell monolayers were washed twice with Dulbecco’s phosphate-buffered saline (DPBS; PAN Biotech GmbH, Aidenbach, Germany) and HEV (2.4 × 10^6^ genome copies/mL; 200 µL/well for 24-well plates; 90 µL/well of a ten-fold dilution series for 96-well plates) was added. After incubation for 1 h at room temperature, the virus solution was removed and the cells were washed three times with PBS. Cell culture medium containing 5% FCS was added. Cells were incubated for seven days at 34.5 °C and 5% CO_2_ and the supernatant was exchanged by fresh medium containing 5% FCS followed by an additional incubation for seven days under the same conditions. The time-course analyses of HEV replication in A549, A549/D3 and A549/DB3 cells were performed in 6-well plates using similar conditions as above (infection with 5.6 × 10^6^ genome copies/mL; 500 µL /well for infection), but removing half of the medium every day and replacing it by the same amount of fresh medium. The cells were investigated by immunofluorescence test (IFT); cell culture supernatants were stored at −20 °C and analyzed by RT-qPCR.

### 2.5. RT-qPCR

In laboratory 1, which performed the initial tests of different established cell lines, RNA was isolated from the culture supernatants on an EZ1^®^ Advanced XL workstation using the EZ1 Virus Mini Kit v2.0 (Qiagen, Hilden, Germany) and tested by RT-qPCR according to Wenzel et al. [14]. HEV RNA concentrations determined by this method are shown as genome copies per mL and the detection limit of the assay was 25 genome copies. One genome copy is equivalent to 0.586 World Health Organization (WHO) units (as defined by the 1st WHO International Standard for HEV NAT Assays, PEI code 6329/10). In laboratory 2, which performed all other experiments, RNA was isolated using the Nuclisense EasyMag (bioMérieux, Marcy l’Etoile, France) and a real-time RT-qPCR [15] (detection limit: 68 genome copies) was performed and quantified as described by Schielke et al. [7].

### 2.6. Immunofluorescence Test 

The IFT was performed as described recently [16]. Briefly, the cells were fixed in acetone/methanol, dried, washed with PBS and blocked with PBS supplemented with 1% FCS. A HEV capsid protein-specific rabbit hyperimmune serum [16] and fluorescein isothiocyanate (FITC)-conjugated anti-rabbit immunoglobulin G (IgG; Sigma, Deisenhofen, Germany) were used for HEV detection. Cells were analyzed using a Zeiss Axio Observer Z1 microscope (Zeiss, Jena, Germany).

### 2.7. Transcriptome Analysis

Cells were grown in T25 flasks at conditions as described above. At the time-point of inoculation, three flasks of each cell line (A549, A549/D3) were removed without infection for transcriptome analysis. Additional three flasks were inoculated with HEV, which served as controls and were later tested for virus replication by RT-qPCR. The non-infected cells were washed three times with PBS and the cellular RNA was isolated using the RNeasy Mini Kit (Qiagen). The RNA of the three replicates was mixed and subjected to transcriptome analysis using the Affymetrix Human Gene 2.0 ST Array (Affymetrix, Santa Clara, CA, USA). The transcriptome and data analysis was performed by ATLAS Biolabs GmbH (Berlin, Germany).

### 2.8. Antibody Blocking Assay

Cells were seeded into 24-well plates and grown for seven days as described above. The medium was exchanged and cells were incubated at 37 °C for additional three days. After two washing steps with PBS, 200 µL/well MEM Eagle medium supplemented with 10 µg of the respective specific rabbit hyperimmune serum was added. CEACAM1-specific (Catalog No. ABIN 1997567), CEACAM5-specific (Catalog No. ABIN 1998195) and CEACAM6-specific (Catalog No. ABIN 1997583) sera were purchased from antibodies-online GmbH (Aachen, Germany). Syndecan 2 (SDC2)-specific antibodies (Catalog No. R30996) originated from NSJ Bioreagents (San Diego, CA, USA). All specific antisera were previously tested negative for HEV-specific antibodies using an enzyme-linked immunosorbent assay (ELISA; AXIOM, Bürstadt, Germany). Control wells received no antiserum. After incubation for 1 h at 37 °C, the solution was removed and replaced by 200 µL HEV-containing culture supernatant (2.4 × 10^6^ genome copies/mL) and incubated at room temperature for 1 h. The virus solution was removed, the cells were washed three times with PBS and culture medium containing 10 µg of the respective antiserum and 5% FCS was added. After incubation for seven days at 34.5 °C, the culture supernatant was removed and replaced by fresh medium containing 10 µg of the respective antiserum and 5% FCS. After additional incubation for seven days at 34.5 °C, RNA was extracted from the culture supernatants and tested by RT-qPCR.

## 3. Results

### 3.1. Inoculation of Different Cell Lines with HEV Strain 47832c

**Figure 1:**
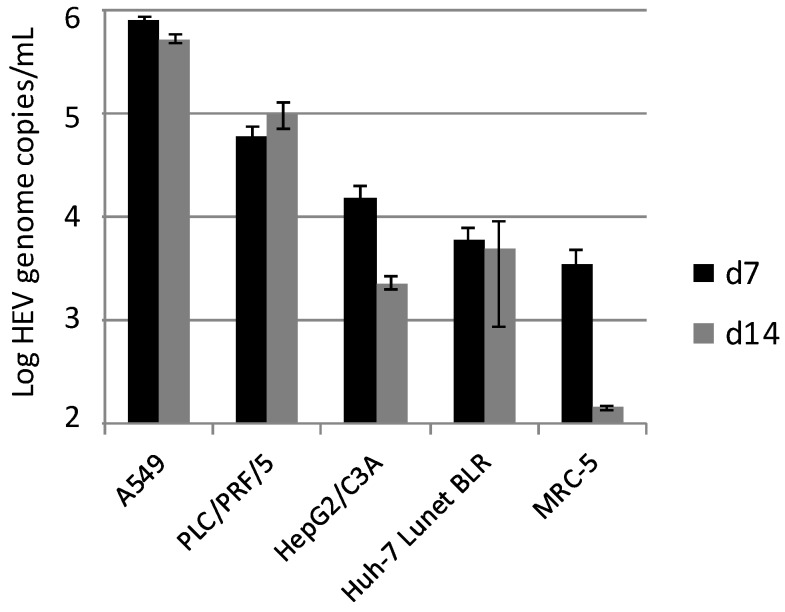
Replication of hepatitis E virus (HEV) strain 47832c in different cell lines. The mean HEV genome copy number present in the culture supernatant at 7 and 14 days (d) after inoculation and the standard deviation (error bars) of three replicates each are shown. For MRC-5 cells at day 14 after inoculation, HEV-RNA was only detectable in two of the three replicates.

The human liver carcinoma cell lines PLC/PRF/5, HepG2/C3A and Huh-7 Lunet BLR, as well as the lung fibroblast cell line MRC-5 and the human lung carcinoma cell line A549 were inoculated with the HEV strain 47832c. Culture supernatants were analyzed at 7 and 14 days p.i. by RT-qPCR. As shown in Figure 1, the highest amounts of the HEV genome were detected in A549 cells and—with an approximately 5-fold lower amount—in PLC/PRF/5 cells. In contrast, HepG2/C3A, Huh-7 Lunet BLR and MRC-5 showed only marginal signs of HEV replication, and decreasing HEV genome amounts between days 7 and 14 p.i. in HepG2/C3A and MRC-5 cells. Based on these results, A549 cells were selected for further experiments to optimize the HEV cell culture system.

### 3.2. Generation and HEV Inoculation of A549-Derived Subclonal Cell Lines

**Figure 2:**
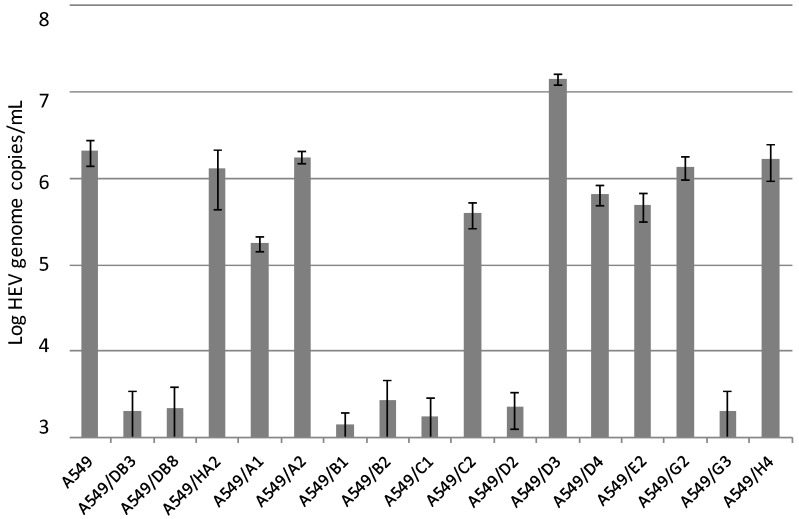
Replication of HEV strain 47832c in clonal cell lines derived from single A549 cells. The HEV genome copy number present in the culture supernatant at 14 days after infection and the standard deviation (error bars) of three replicates each are shown.

Subclonal cell lines were generated by seeding and subsequent growing of single cells of the cell line A549. From two independent experiments, 16 subclonal cell lines were selected and inoculated with the HEV strain 47832c. Analysis of the culture supernatants at 14 days p.i. showed similar or lower amounts of the HEV genome as compared to the parental cell line A549 for 15 of the clonal cell lines, but an approximately 8-fold higher amount for the subclonal cell line A549/D3 (Figure 2).

This result was confirmed by a separate testing of virus growth in A549/D3 cells and A549 cells using the slightly modified protocol used in laboratory 1 (data not shown).

**Figure 3:**
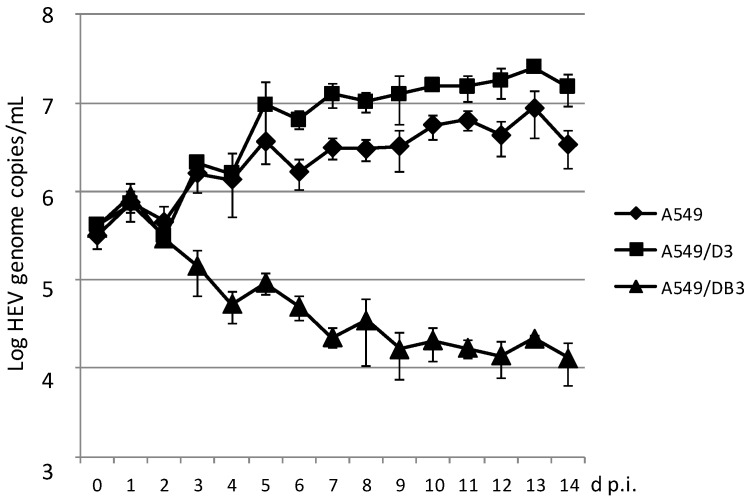
Time-course analysis of HEV strain 47832c replication in A549 cells and in the clonal cell lines A549/D3 and A549/DB3. The HEV genome copy number present in the culture supernatant was analyzed daily and the mean copy number and the standard deviation (error bars) of three replicates each are shown.

A time course analysis with daily measurement of the HEV genome copy number in the culture supernatants was performed for the cell line A549 and for the subclonal cell lines A549/D3 and A549/DB3. As evident from Figure 3, the amount of HEV RNA increased in A549 cells and A549/D3 cells during the first week after infection and remained thereafter at a plateau. Beginning with day 5 p.i., the A549/D3 cells produced higher HEV RNA amounts as compared to the original A549 cells. In contrast, the HEV RNA amounts in the supernatant of cell line A549/DB3 continuously declined during this experiment. HEV RNA observed in all cases between day 0 and 2 days p.i. may represent passive detachment of virus particles bound to the cells.

**Figure 4:**
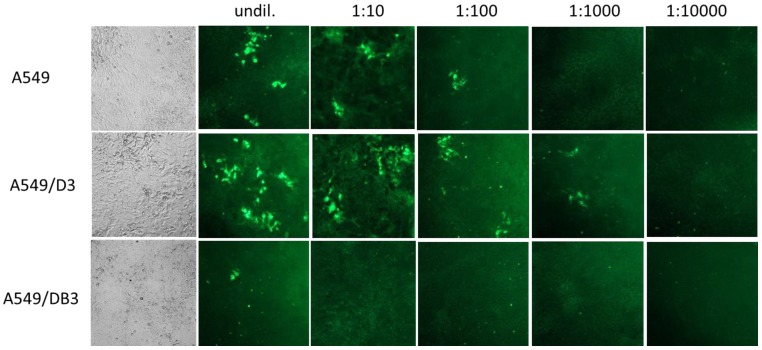
Microscopic analysis of A549 cells and the clonal cell lines A549/D3 and A549/DB3 at 14 days after infection with HEV strain 47832c. Left column: Light microscopic analysis by phase contrast. Other columns: Immunofluorescence analysis of cells infected with different dilutions of an HEV suspension using an HEV capsid protein-specific antiserum.

Light microscopy of the cell line A549 and the subclonal cell lines A549/D3 and A549/DB3 showed that they have similar morphologies; however, A549/D3 cells tended to be larger and showed a more structured appearance (Figure 4, left panel). No cytopathic effects were evident after HEV infection for each of the cell lines. Replication of HEV in these cell lines was also analyzed by IFT using an HEV capsid protein-specific antiserum. To this end, the cell lines were infected with ten-fold dilutions of HEV strain 47832c and analyzed at 14 days p.i. Productive HEV infection was evident in case of cell lines A549 and A549/D3 by the presence of fluorescent foci consisting of more than two adjacent cells showing clear cytoplasmatic fluorescence (Figure 4). These foci were consistently present after infection with virus dilutions of up to 1:100 in the cell line A549 and for 1:1000 dilutions in cell line A549/D3. In contrast, only single isolated cells showed fluorescence after infection of A549/DB3 cells with the undiluted virus suspension.

### 3.3. Transcriptome Analysis of Cells

RNA derived from A549 cells and A549/D3 cells at the time point immediately before infection were analyzed for the transcription of genes using an Affymetrix Human Gene 2.0 ST Array. Table 1 shows the genes with the highest degree of upregulation and downregulation in A549/D3 cells as compared to A549 cells. The most evident difference between both cell lines was a 50-fold downregulation of the gene encoding the *CEACAM6* in the A549/D3 cells. Another gene of a member of the same protein family, *CEACAM 5*, was 21-fold downregulated in the A549/D3 cells. In addition, the *SDC2* gene was 11-fold upregulated in A549/D3 cells.

A similar transcriptome analysis was performed with the subclonal cell line A549/DB3 compared to A549 cells. The genes showing the highest up- and downregulation in A549/DB3 cells are different from that identified in A549/D3 cells (Table S1).

### 3.4. Effect of Anti-CEACAM and Anti-SDC2 Antibodies on HEV Replication in A549/D3 and A549 Cells

**Figure 5:**
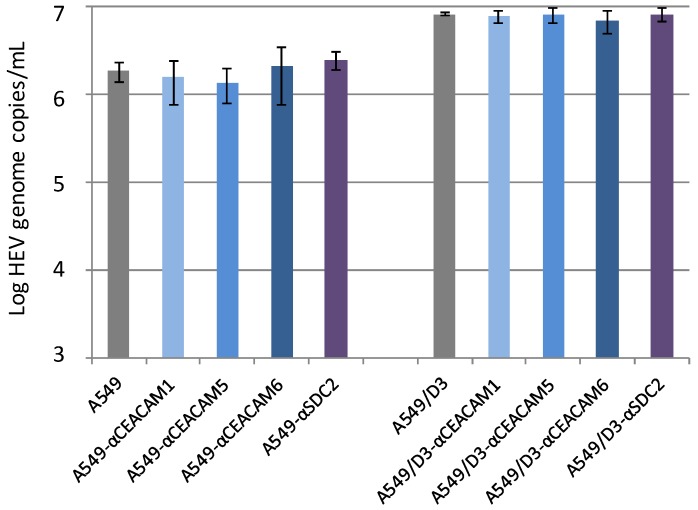
Effect of treatment of cells with carcinoembryonic antigen-related cell adhesion molecule (CEACAM)- and syndecan 2 (SDC2)-specific antibodies on the replication of HEV strain 47832c. A459 cells and A549/D3 cells were incubated with or without CEACAM1-, 5-, 6- and SDC2-specific antisera prior and after HEV inoculation. The HEV genome copy number present in the culture supernatant at 14 days after infection and the standard deviation (error bars) of three replicates each are shown.

As the products of the *CEACAM* and *syndecan* gene families are membrane-bound surface proteins, a blocking of them with antibodies, which possibly prevent the interaction with HEV, was analyzed. To this end, A549 cells and A549/D3 cells were incubated with CEACAM1-, 5- and 6- as well as SDC2-specific antibodies before inoculation with HEV strain 47832c. These antibodies were also supplemented in the culture medium after inoculation. Cells infected and incubated in the absence of specific antibodies served as negative controls. As evident from Figure 5, no significant differences could be found in both cell lines between HEV genome copy numbers in the culture supernatant at 14 days p.i. in the presence or absence of the specific antibodies.

## 4. Discussion

Although the isolation of several HEV strains in different cell culture systems has been described repeatedly, their replication is mostly slow, inefficient and requires large amounts of inoculum. However, efficient cell culture systems for HEV are urgently needed to investigate its replication cycle as well as neutralization and inactivation properties. Therefore, the growth of an already cell culture-isolated HEV strain should be optimized in this study. The strain 47832c, originally isolated from a chronically infected transplant patient, was selected as it belongs to the currently emerging genotype 3 of HEV and showed robust cell culture replication in previous studies [8]. The strain contains a specific genome insertion within the hypervariable region of its ORF1, which may be involved in stable replication in cell culture. As only this strain has been tested in this study, it remains unclear whether other strains show the same replication properties in the cell lines analyzed here.

In a first attempt of optimization, several human cell lines have been tested. The liver cell lines PLC/PRF/5, HepG2/C3A and Huh-7 have been already described to support HEV replication [4,6,17]. In our experiments, however, only PLC/PRF/5 led to efficient virus replication. Although the liver cell is suspected to be the main target of HEV, the specific requirements for HEV replication in cultured liver cells are not yet known. The fetal lung cell line MRC-5 only marginally supported HEV replication, whereas the cell line A549 derived from lung carcinoma cells showed the best replication efficiency. A549 cells have been repeatedly described in successful HEV isolation experiments [18,19,20]; also, strain 47832c has been originally isolated in this cell line. The distinct properties of this cell line leading to HEV susceptibility should be investigated in more detail.

Based on a hypothesis that the A549 cell line is a mixture of different cell types, subclonal cell lines were generated. The fact that the subclonal cell lines showed marked differences in HEV replication efficiency confirms this hypothesis and underlines the heterogeneity of A549 cells. In this context, it should also be considered that the A549 cell lines used in different laboratories may be heterogeneous and therefore show different efficiencies of HEV replication. Subclonal cell lines with differing efficiency of HEV replication have also been described very recently for the PLC/PRF/5 cell line [21], thus indicating that this phenomenon is not unique for A549 cells. The selected subclonal cell lines may later be used in improved HEV cell culture systems. The resulting subclonal cell line A549/D3 has already been applied in a titration system for testing the heat stability of HEV [16].

In order to investigate the underlying mechanism of enhanced HEV replication, a transcriptome analysis was performed for the subclonal cell line A549/D3. Among others, this led to the identification of *CEACAM* genes, which encode membrane-bound members of the immunoglobulin superfamily, overexpressed in many cancers and associated with adhesion and invasion [22,23]. In addition, CEACAM6 was reported to attenuate the adenovirus infection in cancer cells by antagonizing intracellular trafficking [24]. In order to investigate if these molecules bind HEV and thereby prevent its replication in A549 cells compared to A549/D3 cells, an antibody blocking experiment was performed. Another identified gene of a membrane-bound protein was the *SDC2* gene, which was upregulated in A549/D3 cells. Syndecans are heparan sulfate proteoglycans [25], which already have been shown to be capable of HEV capsid protein binding [26] and have been found as a potential Dengue virus receptor in vitro [27]. Therefore, blocking experiments should indicate if these molecules are responsible for enhanced uptake of HEV into A549/D3 cells followed by increased replication. However, no effect either of the CEACAM-specific antibodies or of the SDC2-specific antibodies could be found in the experiments presented here. Therefore, either these molecules are acting by more indirect mechanisms or their up- or downregulation is not the cause of the improved HEV replication in A549/D3 cells. In a similar analysis of A549/DB3 cells, other genes were up- or downregulated, indicating that a different mechanism leads to the very low HEV replication activity of these cells. Very recently, the asialoglycoprotein receptor has been identified as a molecule facilitating HEV infection in PLC/PRF/5 cells [28]. However, no differences could be found in the expression levels of this gene between A549 and A549/D3 or A549/DB3 cells, thus excluding this molecule as the cause of different replication efficiency in these cell lines. Further experiments should also include transcriptome analyses of HEV-infected cells as HEV may modulate cellular gene expression during its replication as recently shown for antiviral innate immunity pathways [29,30].

In conclusion, the cell culture system for HEV was improved by the generation of the clonal cell line A549/D3, which now can be used for the investigation of the HEV replication cycle and its neutralization and inactivation properties. Further improvements of the system may be achieved by testing different cell culture conditions and supplements. In addition, further investigations should be initiated to unravel the mechanism of improved replication in these cells. The identification of specific cellular factors supporting HEV replication would allow a more targeted improvement of HEV cell culture systems and provide general insights into the virus-host cell interplay during HEV infection.

## Figures and Tables

**Table 1:** Up- and downregulated genes in A549/D3 cells compared to A549 cells.

Probe ID	Gene Symbol	Gene Name	Fold Change
Upregulated			
17071144	*SDC2*	*Syndecan 2*	11.005
17107867	*MAGEA6/3*	*Melanoma antigen family A 6 and 3*	8.843
17077525	*CA8*	*Carbonic anhydrase VIII*	7.308
17023338	*RNA5SP216*	*RNA, 5S ribosomal pseudogene 216*	7.219
16948063	*NLGN1*	*Neuroligin 1*	6.374
Downregulated			
16862563	*CEACAM6*	*Carcinoembryonic antigen-related cell adhesion molecule 6*	−50.609
16872621	miscellaneous	Chromosome (Chr) 19: 42219580–42223939	−23.484
16862548	*CEACAM5*	*Carcinoembryonic antigen-related cell adhesion molecule 5*	−21.642
16738803	*TCN1*	*Transcobalamin I*	−18.276
16879863	*EPCAM*	*Epithelial cell adhesion molecule*	−13.057

## Supplementary Materials

The following are available online at http://www.mdpi.com/1999-4915/8/10/267/s1.
