# Supplementary material for: Enhanced Replication of Hepatitis E Virus Strain 47832c in an A549-Derived Subclonal Cell Line
Source: Viruses. 2016 Sep 29;8(10):267. doi: 10.3390/v8100267 (PMC5086603; doi:10.3390/v8100267)
Supplement: Supplementary file 1 [file viruses-08-00267-s001.pdf]

Supplementary Materials: Enhanced Replication of Hepatitis E Virus Strain 47832c in an A549-Derived Subclonal Cell Line

Mathias Schemmerer, Silke Apelt, Eva Trojnar, Rainer G. Ulrich, Jürgen J. Wenzel and Reimar Johne

Table S1. Up- and downregulated genes in A549/DB3 cells compared to A549 cells.

Probe ID	Gene Symbol	Gene Name	Fold Change
Upregulated			
16944325	*UPK1B*	*Uroplakin 1B*	15.350
16942866	*CADM2*	*Cell adhesion molecule 2*	14.694
16817154	*SLC5A11*	*Solute carrier family 5, member 11*	13.982
16798938	*SCG5*	*Secretogranin V*	11.461
16911493	*SPTLC3*	*Serine palmitoyltransferase, long chain base subunit 3*	9.852
Downregulated			
16960922	*RARRES1*	*Retinoic acid receptor responder (tazarotene induced) 1*	−14.993
16712576	*PRTFDC1*	*Phosphoribosyl transferase domain containing 1*	−14.523
16974830	*PPARGC1A*	*Peroxisome proliferator-activated receptor gamma, coactivator 1 alpha*	−12.992
17059955	*PDK4*	*Pyruvate dehydrogenase kinase, isozyme 4*	−12.904
16795908	*CATSPERB*	*Catsper channel auxiliary subunit bets*	−9.446
